# On gaps of clinical diagnosis of dementia subtypes: A study of Alzheimer’s disease and Lewy body disease

**DOI:** 10.3389/fnagi.2023.1149036

**Published:** 2023-03-21

**Authors:** Hui Wei, Arjun V. Masurkar, Narges Razavian

**Affiliations:** ^1^Manning College of Information and Computer Sciences, University of Massachusetts Amherst, Amherst, MA, United States; ^2^Department of Population Health, NYU Grossman School of Medicine, New York, NY, United States; ^3^Center for Cognitive Neurology, Department of Neurology, NYU Grossman School of Medicine, New York, NY, United States; ^4^Neuroscience Institute, NYU Grossman School of Medicine, New York, NY, United States; ^5^Department of Radiology, NYU Grossman School of Medicine, New York, NY, United States; ^6^Center for Data Science, New York University, New York, NY, United States

**Keywords:** Alzheimer’s disease, Lewy body disease, clinical diagnosis accuracy, autopsy-confirmed results, dementia stages, disparity

## Abstract

**Introduction:**

Alzheimer’s disease (AD) and Lewy body disease (LBD) are the two most common neurodegenerative dementias and can occur in combination (AD+LBD). Due to overlapping biomarkers and symptoms, clinical differentiation of these subtypes could be difficult. However, it is unclear how the magnitude of diagnostic uncertainty varies across dementia spectra and demographic variables. We aimed to compare clinical diagnosis and post-mortem autopsy-confirmed pathological results to assess the clinical subtype diagnosis quality across these factors.

**Methods:**

We studied data of 1,920 participants recorded by the National Alzheimer’s Coordinating Center from 2005 to 2019. Selection criteria included autopsy-based neuropathological assessments for AD and LBD, and the initial visit with Clinical Dementia Rating (CDR) stage of normal, mild cognitive impairment, or mild dementia. Longitudinally, we analyzed the first visit at each subsequent CDR stage. This analysis included positive predictive values, specificity, sensitivity and false negative rates of clinical diagnosis, as well as disparities by sex, race, age, and education. If autopsy-confirmed AD and/or LBD was missed in the clinic, the alternative clinical diagnosis was analyzed.

**Findings:**

In our findings, clinical diagnosis of AD+LBD had poor sensitivities. Over 61% of participants with autopsy-confirmed AD+LBD were diagnosed clinically as AD. Clinical diagnosis of AD had a low sensitivity at the early dementia stage and low specificities at all stages. Among participants diagnosed as AD in the clinic, over 32% had concurrent LBD neuropathology at autopsy. Among participants diagnosed as LBD, 32% to 54% revealed concurrent autopsy-confirmed AD pathology. When three subtypes were missed by clinicians, “No cognitive impairment” and “primary progressive aphasia or behavioral variant frontotemporal dementia” were the leading primary etiologic clinical diagnoses. With increasing dementia stages, the clinical diagnosis accuracy of black participants became significantly worse than other races, and diagnosis quality significantly improved for males but not females.

**Discussion:**

These findings demonstrate that clinical diagnosis of AD, LBD, and AD+LBD are inaccurate and suffer from significant disparities on race and sex. They provide important implications for clinical management, anticipatory guidance, trial enrollment and applicability of potential therapies for AD, and promote research into better biomarker-based assessment of LBD pathology.

## 1. Introduction

Alzheimer’s disease (AD) is the most common cause of dementia, accounting for 60–80% of dementia cases (Scheltens et al., 2016; Soria Lopez et al., 2019). AD is neuropathologically defined by amyloid plaque and neurofibrillary tau tangles, and typically presents clinically as a progressive amnestic syndrome with minimal physical symptoms, especially in the early stages. Lewy body disease (LBD) is the second most prevalent neurodegenerative dementia subtype (Walker et al., 2015; Mueller et al., 2017). In contrast to AD, LBD is neuropathologically linked to alpha synuclein-based Lewy body inclusions. It is clinically hallmarked by cognitive fluctuations, early onset visual hallucinations, REM sleep behavior disorder, and parkinsonism, with supportive features including atypical response to neuroleptics, autonomic dysfunction, and dysosmia (McKeith et al., 2017).

Yet despite these seemingly disparate classical descriptions, in clinical practice there can be significant overlap between their presentations. AD and LBD can have similar neuropsychological profiles, especially considering dysexecutive and other variants of AD. Hallucinations tend to occur later in AD but can sometimes appear at early stages. Parkinsonism can develop in the late stages of AD as well (Morris et al., 1989; Huang and Halliday, 2013). Dysosmia is found in both AD and LBD. Lastly, *APOE4* has been found to influence pure LBD pathology independent of AD pathology (Dickson et al., 2018). Moreover, AD and LBD can be found together on autopsy. This mixed subtype (AD+LBD) can lead to a more severe presentation (Hansen, 1997; Förstl, 1999) with symptoms of both manifesting concurrently. Available clinical diagnostic criteria for AD (McKhann et al., 2011) and LBD (McKeith et al., 2005) perform poorly when applied to this mixed dementia. How to identify and treat this mixed subtype is still an open research area, especially as there are biomarker-based assessments for AD but not LBD.

Inaccurate or delayed recognition of dementia subtypes can have significant impact on clinical care. Recognition of an LBD component (LBD or AD+LBD) would suggest more caution with neuroleptic use. Knowledge of an LBD component would also permit anticipatory guidance for patients and families regarding the potential for motoric decline, psychosis, dysautonomia, and REM sleep behavior disorder. In the advent of disease-modifying amyloid-lowering therapies, knowing the likelihood of concurrent AD pathology will be important in determining whether such medications would be of value. This same consideration is applicable to ongoing and future clinical trials focusing on AD and LBD.

Several previous studies assessed clinical diagnostic quality for dementia subtypes. (Beach et al., 2012) examined the clinical AD diagnosis accuracy of 919 participants at their last assessment during life from the 2005–2010 National Alzheimer’s Coordinating Center dataset. They concluded the clinical diagnosis is more accurate for AD than non-AD dementia. (Soria et al., 2018) found the same result for 53 Latino participants in UCSD Alzheimer’s Disease Research Center dataset from 1991 to 2017. Similarly, (Selvackadunco et al., 2019) measured the mismatch rate between clinical diagnosis and post-mortem neuropathological results of 7 dementia subtypes for 180 participants in the Brains for Dementia Research cohort. They found the clinical misdiagnosis rate is high among all 7 subtypes. Gianattasio et al. (2019) and Lin et al. (2020) evaluated the racial dementia diagnosis disparity and found that Non-Hispanic Black participants have a higher underdiagnosis rate compared to Non-Hispanic White participants. Yet, these studies did not differentiate dementia stages or only focused on later stages. These results motivate a deeper analysis of diagnostic quality and disparity at all dementia stages separately to assess clinical diagnosis quality at earlier stages and whether it improves at higher stages.

In this paper, we focus on autopsy-confirmed diagnosis of 1,920 dementia participants, and assess the clinical diagnosis made for them at different stages of their cognitive impairment (CDR 0.5, 1.0, 2.0, and 3.0). We focus on the sensitivity, specificity, and positive predictive value of AD, LBD, and AD+LBD subtypes, as well as disparity measures. Our results demonstrate that the current clinical diagnosis accuracy is still low at all cognitive impairment stages for all three dementia subtypes, and there are significant disparities based on race and sex. These findings have important implications for clinical enrollments for both AD and LBD-specific pathologies as well as clinical care. They also highlight the value of developing sensitive biofluid or imaging-based biomarkers for LBD.

## 2. Materials and methods

### 2.1. Study design and participants

**FIGURE 1:**
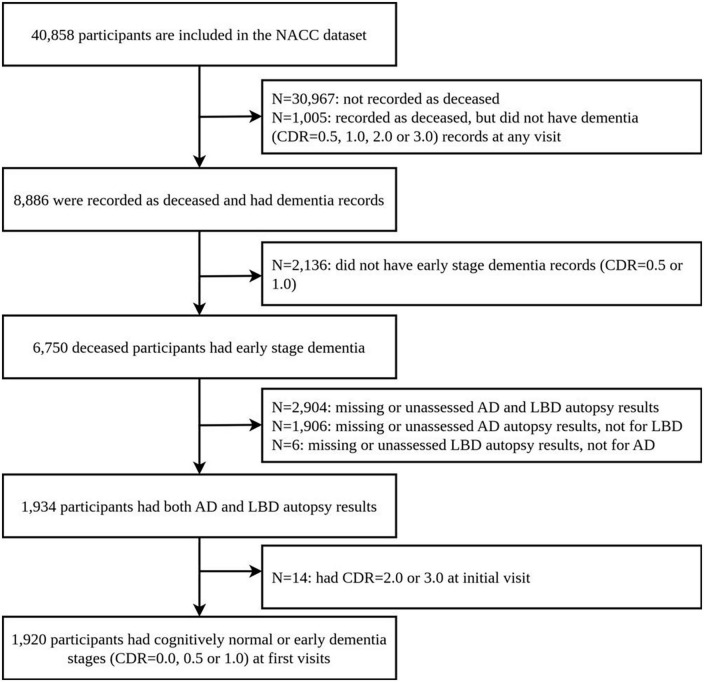
Flow of participants.

We retrospectively selected participants from clinical case series at the Alzheimer’s Disease Centers (ADCs) from the National Alzheimer’s Coordinating Center (NACC), using longitudinal Uniform Data Set (UDS) and the Neuropathology (NP) Data Set, collected up to 20 August 2019. Their autopsy results and clinical diagnosis results are freely available. This data includes records from 40,858 participants, 1,934 of whom had non-missing post-mortem brain autopsy results for AD and region-specific LBD pathologies. Neuropathology data was limited to participants who had completed NACC Neuropathology from version 10, as earlier versions did not collect the data using the latest definitions we used. We further limit our analysis to participants: (1) who had clinical evaluation and diagnosis for AD and/or LBD and (2) whose first record in the NACC data shows cognitively normal (CDR 0.0) or early dementia stages (CDR 0.5 or 1.0). This results in 1,920 participants in the final study. Figure 1 shows the flow of participants, and the basic information of the dataset is also presented in Supplementary Table 1 of the Supplementary material.

### 2.2. Autopsy-confirmed output definition

In our analysis, we define a participant outcome as AD, LBD, AD+LBD, or neither. We note that in this paper we do not focus on other dementia subtypes such as vascular dementia (VD) or frontotemporal lobar degeneration (FTLD). Definitions of each autopsy-confirmed subtype in our study are as follows.

**AD:** Participants has undergone autopsy and their NIA-AA Alzheimer’s disease neuropathologic change, ADNC-ABC score (Hyman et al., 2012)^1^ is 2 (intermediate) or 3 (high), and their Lewy body pathology derived result is marked as 0 (No Lewy body pathology) or 4 (Lewy bodies present, but region unspecified or found in the olfactory bulb).

**LBD:** Participants has undergone autopsy and their NIA-AA Alzheimer’s disease neuropathologic change, ADNC-ABC score is either 0 (Not AD) or 1 (Low ADNC), and their Lewy body pathology derived results^2^ is marked as 1 (Brainstem—predominant), 2 (Limbic—transitional), or 3 (Neocortical—diffuse).

**AD + LBD:** Participants has undergone autopsy and their NIA-AA Alzheimer’s disease neuropathologic change (ADNC) (ABC score) is 2 (intermediate) or 3 (high); and their Lewy body pathology derived results is marked as 1 (Brainstem—predominant), 2 (Limbic—transitional) or 3 (Neocortical—diffuse).

**Neither:** Participants who have undergone autopsy and do not match any of the AD, LBD, or AD+LBD criteria.

### 2.3. Clinical diagnosis identification

We define clinical diagnosis as either AD, LBD, AD+LBD, or neither. The clinical definitions are as follows:

**AD:** Clinician has marked the diagnosis as *Presumptive etiologic diagnosis of the cognitive disorder—Alzheimer’s disease^3^*; and the *Presumptive etiologic diagnosis of the cognitive disorder—Lewy body disease^4^* is marked as 0 (No).

**LBD:** Clinician has marked the diagnosis as *Presumptive etiologic diagnosis of the cognitive disorder—Lewy body disease^5^*, and the *Presumptive etiologic diagnosis of the cognitive disorder—Alzheimer’s disease^6^* is marked as 0 (No).

**AD + LBD:** Both positive diagnosis of *Presumptive etiologic diagnosis of the cognitive disorder—Alzheimer’s disease* and *Presumptive etiologic diagnosis of the cognitive disorder—Lewy body disease* are recorded^7^.

**Neither:** If none of the criteria above is met.

### 2.4. Evaluation time points

We evaluate the clinical diagnosis accuracy for the included participants at Cognitive Dementia Rating Global Score (Morris, 1993) (CDR) of 0.5, 1.0, 2.0, and 3.0. At each cognitive impairment level, we evaluate clinical diagnosis accuracy at the first visit where the CDR score becomes equal to 0.5, 1.0, 2.0, or 3.0. We compare the clinical diagnosis against the autopsy results.

### 2.5. Statistical analysis and evaluation metrics

All our results include confidence intervals, measured via bootstrapping method. Our bootstrapping algorithm includes 1,000 iterations, and results are reported with 95% confidence intervals. *P*-values are computed based on one-sided testing and a 0.05 level indicates significance.

For evaluating accuracy of clinical diagnosis, we report positive predictive value, sensitivity, specificity and false negative rates, as well as confusion matrix between clinical diagnosis and autopsy results at various dementia stages (CDR 0.5, 1.0, 2.0, and 3.0). For those participants clinically diagnosed as *Neither* at different dementia stages, we further report the primary etiologic diagnosis^8^ made by the clinician instead.

### 2.6. Diagnosis disparities

We also measure clinical diagnosis disparities based on sex, race, age, education years and cognitive decline durations^9^ at visits. We report, per subgroup, the average metrics over all subtypes (if one subtype is missing, the average is computed based on other non-missing subtypes). Thus, the bootstrapping results show the distribution of average diagnosis performances. Besides metrics used in Table 3, we also compute false negative rate (FNR) for each group.

## 3. Results

Overall, 1,920 participants met the criteria for inclusion, of which 788 had autopsy-confirmed AD, 140 had autopsy-confirmed LBD, 572 had autopsy-confirmed AD+LBD, and 420 were confirmed to have neither. Table 1 includes the characteristics for these participants. Participants who have LBD during autopsy are more likely to be male (66% vs. 56% *p*-value: 0.02). Participants with AD+LBD at autopsy have overall higher CDR at first visits (0.66 vs. 0.59 *p*-value: <0.001).

**TABLE 1:** Participants characteristics.

Characteristics	All participants	Autopsy confirmed Alzheimer’s disease	Autopsy confirmed Lewy body disease	Autopsy confirmed AD+LBD	Neither AD nor LBD confirmed at autopsy
**Total number of included participants**	1920	788	140	572	420
**Sex**					
Male	1071 (56%)	403 (51%)	92 (66%)	326 (57%)	250 (60%)
Female	849 (44%)	385 (49%)	48 (34%)	246 (43%)	170 (40%)
**Race**					
White	1796 (94%)	734 (93%)	135 (96%)	537 (94%)	390 (93%)
Black or African American	58 (3.0%)	25 (3.17%)	0 (0.00%)	18 (3.15%)	15 (3.57%)
American Indian or Alaska Native	2 (0.10%)	2 (0.25%)	0 (0.00%)	0 (0.00%)	0 (0.00%)
Native Hawaiian or Pacific Islander	1 (0.05%)	0 (0.00%)	0 (0.00%)	0 (0.00%)	1 (0.24%)
Asian	14 (0.73%)	4 (0.51%)	0 (0.00%)	5 (0.87%)	5 (1.19%)
Multiracial	36 (1.88%)	17 (2.16%)	5 (4%)	9 (1.57%)	5 (1.19%)
Unknown or ambiguous	13 (0.68%)	6 (0.76%)	0 (0.00%)	3 (0.52%)	4 (0.95%)
**# of participants at each CDR stage**					
CDR 0.5	1262	524	108	326	304
CDR 1.0	1368	589	82	461	236
CDR 2.0	831	373	37	298	123
CDR 3.0	609	266	26	212	105
**# of participants at each age stage**					
<60	196	68	8	57	63
60–70	485	173	34	142	136
70–80	742	309	53	277	103
80–90	738	351	53	221	113
>=90	236	118	13	42	63
**# of participants at each education level (in education years)**					
<=12	377 (19.64%)	162 (20.56%)	30 (21.43%)	102 (17.83%)	83 (19.76%)
12–16	852 (44.38%)	365 (46.32%)	55 (39.29%)	253 (44.23%)	179 (42.62%)
>16	674 (35.10%)	257 (32.61%)	55 (39.29%)	213 (37.24%)	149 (35.48%)
Unknown	17 (0.89%)	4 (0.51%)	0 (0.00%)	4 (0.70%)	9 (2.14%)
**Mean [STD] age at initial visits**	73.62 [10.53]	75.16 [9.94]	74.49 [9.24]	72.83 [9.54]	71.53 [12.63]
**Mean [STD] CDR at initial visits**	0.59 [0.35]	0.59 [0.35]	0.52 [0.34]	0.66 [0.34]	0.51 [0.37]
**Mean [STD] education years**	15.67 [3.03]	15.56 [3.02]	15.64 [3.27]	15.88 [3.04]	15.59 [2.96]

The number in the parenthesis is the ratio between the number of participants with that characteristic and the number of all participants or participants with autopsy-confirmed AD or/and LBD results. The number in the bracket is the standard deviation (STD) for participants of that characteristic. Each participant can contribute different data points to different visits with different ages, so the sum of the numbers of participants at each age stage might be larger than the total number of included participants (row 1) of each column. Same case for the number of participants at each CDR stage.

### 3.1. Evaluation of clinical diagnosis

Of 1,920 participants studied, 1,262 had a clinical visit at CDR 0.5; 1,368 participants had a clinical visit at CDR 1.0; 831 participants had a clinical visit at CDR 2.0; and 609 participants had a clinical visit at CDR 3.0.

We analyzed each dementia stage independently and measured the accuracy of the clinical diagnosis for the first visit at each CDR-defined stage. Table 2 includes the distribution of clinical diagnosis results compared to the autopsy results. Table 3 includes measures of positive predictive value (Precision, PPV), sensitivity and specificity of the clinical diagnosis for each stage of dementia.

**TABLE 2:** Distribution of clinical diagnosis vs. autopsy results at CDR 0.5, 1.0, 2.0, and 3.0.

		Clinical Diagnosis				
		AD	LBD	AD+LBD	Neither	CDR
Autopsy Results	**AD**	298	8	9	209	0.5
	**LBD**	24	28	3	53	
	**AD+LBD**	200	26	9	91	
	**Neither**	99	1	3	201	
	**AD**	507	3	24	55	1.0
	**LBD**	30	26	11	15	
	**AD+LBD**	371	33	27	30	
	**Neither**	88	4	1	143	
	**AD**	331	6	11	25	2.0
	**LBD**	9	21	1	6	
	**AD+LBD**	252	14	18	14	
	**Neither**	43	3	4	73	
	**AD**	225	4	13	24	3.0
	**LBD**	6	12	0	8	
	**AD+LBD**	172	19	10	11	
	**Neither**	33	0	3	69	

**TABLE 3:** Clinical diagnosis performance (w/95% CIs) for all dementia stages all subtypes.

Clinical diagnosis	CDR	Precision (PPV)	Sensitivity	Specificity
AD	0.5	0.48 (0.44, 0.52)	0.57 (0.53, 0.62)	0.56 (0.53, 0.60)
	1.0	0.51 (0.48, 0.54)	0.86 (0.83, 0.89)	0.37 (0.34, 0.41)
	2.0	0.52 (0.48, 0.56)	0.89 (0.85, 0.92)	0.34 (0.29, 0.38)
	3.0	0.52 (0.47, 0.57)	0.85 (0.80, 0.89)	0.38 (0.33, 0.44)
LBD	0.5	0.44 (0.32, 0.57)	0.26 (0.18, 0.34)	0.97 (0.96, 0.98)
	1.0	0.39 (0.28, 0.52)	0.32 (0.22, 0.43)	0.97 (0.96, 0.98)
	2.0	0.47 (0.33, 0.62)	0.56 (0.39, 0.72)	0.97 (0.96, 0.98)
	3.0	0.34 (0.19, 0.51)	0.46 (0.27, 0.66)	0.96 (0.94, 0.98)
AD+LBD	0.5	0.37 (0.18, 0.58)	0.03 (0.01, 0.05)	0.98 (0.98, 0.99)
	1.0	0.42 (0.31, 0.55)	0.06 (0.04, 0.08)	0.96 (0.95, 0.97)
	2.0	0.53 (0.37, 0.70)	0.06 (0.04, 0.09)	0.97 (0.95, 0.98)
	3.0	0.38 (0.19, 0.59)	0.05 (0.02, 0.08)	0.96 (0.94, 0.98)
Neither	0.5	0.36 (0.32, 0.40)	0.66 (0.61, 0.72)	0.63 (0.60, 0.66)
	1.0	0.59 (0.53, 0.64)	0.61 (0.54, 0.66)	0.91 (0.89, 0.93)
	2.0	0.62 (0.53, 0.71)	0.59 (0.51, 0.67)	0.94 (0.92, 0.95)
	3.0	0.61 (0.52, 0.71)	0.66 (0.57, 0.75)	0.92 (0.89, 0.94)

**FIGURE 2:**
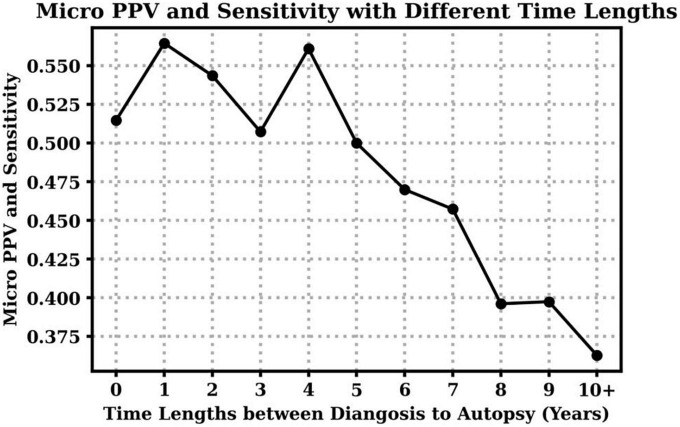
Clinical diagnosis positive predictive values (PPV) and sensitivity with respect to different time lengths between the visit time (clinical diagnosis) to the death time (autopsy). Notice that micro PPV and micro sensitivity are equal based on their definitions in this case.

Additionally, we investigate the impact of time lengths between the clinical diagnosis and the autopsy to clinical diagnosis accuracy over all dementia subtypes. Table 4 shows the mean and standard deviation (std) of time lengths between clinical diagnosis and autopsy at different CDR stages. On average, higher CDR stages have shorter time gaps, which means the clinical diagnoses were closer to the final autopsy time. Figure 2 demonstrates that overall clinical diagnosis accuracy decreases with increased interval between last clinical evaluation and death.

**TABLE 4:** Statistics of time length (in years) from clinical diagnosis to autopsy at different CDR stages.

CDR score	0.5	1.0	2.0	3.0
Time length from clinical diagnosis to autopsy **(Mean [STD])**	6.34 [2.85]	5.04 [2.73]	3.57 [2.30]	2.25 [1.99]

**FIGURE 3:**
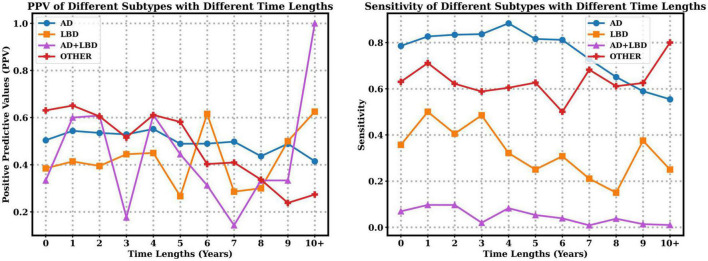
Positive predictive values (PPV) and sensitivity of different subtypes with respect to time lapses from clinical diagnosis time to autopsy time.

We also explore the PPV and sensitivity in a finer granularity with respect to each subtype. Figure 3 indicates that with the increasing time lapse, the clinician diagnosed PPV of AD decreases, but the PPV of LBD increases. Moreover, longer time lapses will lead to lower sensitivity of AD, LBD and AD+LBD, but higher sensitivity of other dementia subtypes. Note that sensitivity of AD+LBD stays extremely low (<0.06) no matter how close the clinician diagnosis time is to autopsy results.

### 3.2. Clinically missed diagnoses

**FIGURE 4:**
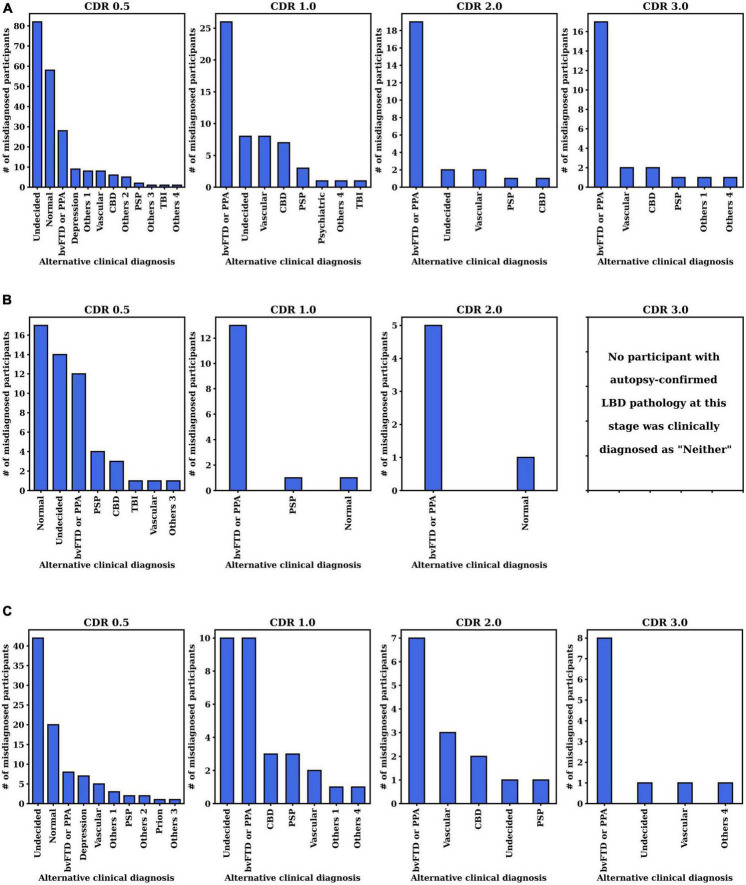
Distribution of clinically primary etiology diagnosis when pathological **(A)** AD, **(B)** LBD, or **(C)** AD+LBD was missed by clinicians. The results are analyzed at the first visits of each CDR stage for each subtype (i.e., AD, LBD, AD+LBD). For the corresponding clinically primary etiology diagnosis names in the NACC dataset, please refer to Supplementary Table 2.

We further investigated the alternative clinical diagnosis made by the clinicians instead of the autopsy-confirmed AD, LBD, or AD+LBD diagnosis (i.e., corresponding to the “Neither” column in Table 2). The histogram of primary clinical etiologic diagnosis made for these cases are included in Figure 4. We report the results at different CDR stages for three autopsy-confirmed subtypes (i.e., AD, LBD, and AD+LBD). Of note, other than AD and/or LBD, the most common alternative clinical diagnoses missing pathological AD, LBD, and AD+LBD were “No cognitive impairment” (Normal) and “behavioral variant frontotemporal dementia (bvFTD) or primary progressive aphasia (PPA)”.

### 3.3. Diagnosis disparities

**FIGURE 5:**
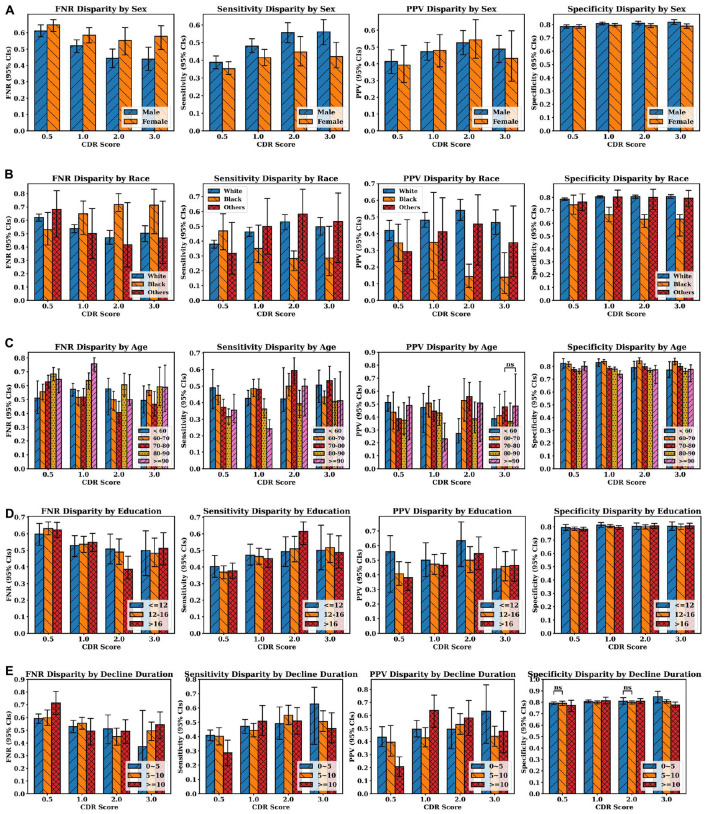
Clinician diagnosis disparities by **(A)** sex, **(B)** race, **(C)** age, and **(D)** education years, **(E)** cognitive decline durations (in years). Disparities are evaluated by false negative rate (FNR), sensitivity, positive predictive values and specificity (from left to right at each row). Error bars indicate 95% confidence intervals (CIs). Independent *t*-test were executed between group pairs at the same CDR stage and the same evaluation metric [e.g., White and Black at CDR 0.5 in FNR disparity plot in panel **(B)**]. If the *p*-value of two groups is larger than 0.05, “ns” (i.e., not significant) is annotated above the corresponding bars. Otherwise, all the *p*-values are less than 0.05, which means the average metric values of two groups are different with statistical significance.

Figure 5 shows the average false negative rate, sensitivity, positive predictive value and specificity across all four classes (AD, LBD, AD+LBD, and Neither) for different subgroups based on sex, race, age, education years, and cognitive decline durations. The average diagnosis quality (both in terms of sensitivity and false negative rate) significantly improves for males as dementia stage progresses, whereas this is not observed for female participants. Except for the questionable impairment stage (CDR 0.5), Black/African-American participants have the lowest clinical diagnosis accuracy across all metrics compared to all other races/ethnicities, which further worsens at more severe dementia stages. Of note, higher education levels do not improve clinical diagnosis accuracy, although we can see a trend of better sensitivity for participants with higher than bachelor’s degree (education years >12) at higher dementia stages. Furthermore, although age does not have a significant impact on clinical diagnosis, the average diagnosis accuracy for participants aged 80–90 years old tends not to be as accurate as other age groups with regard to sensitivity and positive predictive values, especially at later stages. Finally, with increasing dementia stages, the diagnosis accuracy (evaluated by all four metrics) is significantly improved for those participants with cognitive decline durations less than 5 years at the visits. At the early impairment stage (CDR 0.5), participants with decline duration of more than 10 years have the poorest diagnosis quality, compared with other participants who developed the cognitive decline more recently.

## 4. Discussion

Dementia has remained an incurable disease despite the massive burden to patients, their caregivers and society. Despite national investments, clinical trials to find a treatment have not made a breakthrough (Carroll, 2017; Liu et al., 2019; Alexander et al., 2021). Our results indicate that clinical diagnosis has limitations in accurately identifying and distinguishing Alzheimer’s disease, Lewy body disease, and their combination. This has important implications for clinical trials for agents that target β-amyloid clearance (Wirz-Justice, 1986; Kurz and Perneczky, 2011; Ostrowitzki et al., 2017; Honig et al., 2018; Egan et al., 2019; Huang et al., 2020; NIA, 2021; van Dyck et al., 2023) or other subtype-specific neuropathologies. Indeed, we find that of participants diagnosed with AD in the clinic, 32% (CDR 0.5) to 40% (CDR 2.0) had AD+LBD pathology. In the absence of biomarkers for Lewy bodies, using amyloid or tau biomarkers to recruit Alzheimer’s disease participants cannot identify these with AD+LBD. At the moment, clinicians are most likely to miss the concurrent LBD of participants with mixed AD+LBD (missing rate from 61% at CDR 0.5–85% at CDR 2.0). Moreover, the canonical symptoms which are specific to LBD (e.g., visual hallucinations, cognitive fluctuations) at the early stages can be driven by other pathologies, including AD, in later dementia stages, which are closer to autopsy by default. As such, positive predictive values for clinical diagnosis of AD+LBD were low across all stages, ranging from 37 to 53%. Additionally, sensitivity of clinical diagnosis for AD+LBD was poor, ranging from 3% (95% CI: 1%, 5%) at CDR 0.5, to 6% (95% CI: 4%, 9%) at CDR 2.0. And while autopsy-confirmed AD+LBD was more common than LBD (572 vs. 140 overall), this prevalence did not translate to the clinic evaluations. The inaccuracies of clinical diagnosis for AD+LBD have also been recognized in previous smaller studies (Selvackadunco et al., 2019) although regardless of dementia stages. Our finding on much larger data shows that these inaccuracies do not decrease as participants’ impairment increases.

Besides the challenges revealed in clinical diagnosis of AD+LBD, we also find that even Alzheimer’s disease diagnosis is difficult. At early dementia stages (CDR 0.5), clinical diagnosis of AD had low sensitivities 57% (95% CI: 53%, 62%) and specificities 56% (95% CI: 53%, 60%). While sensitivity improved at more advanced dementia stages, specificity further dropped to 38% (95% CI: 33%, 44%) at CDR 3.0. When analyzing non-missing alternative clinical diagnosis made instead of AD, we see “Normal” diagnosis (i.e., no impairment), primary progressive aphasia or behavioral variant frontotemporal dementia were the leading primary etiologic diagnosis when the neuropathological AD was missed. While sensitivity and specificity are important clinically, for clinical trial enrollments, positive predictive values are more important. PPV of clinical diagnosis of AD was only 48% (95% CI: 44%, 52%) at CDR 0.5, and even at higher dementia stages (CDR 3.0), this accuracy remained low at 52% (95% CI: 47%, 57%). This means that of every two participants enrolled in an AD-specific clinical trial, especially those without in vivo biomarker confirmation, one has neuropathologies other than AD, and thus may not experience full benefit from the AD-specific treatments.

Lewy body disease was more commonly manifested as AD+LBD (140 vs 572 in Table 1), which is also suggested by Iseki et al. (1999), and as we found, AD+LBD was often clinically diagnosed as AD only. However, focusing on participants with clinically diagnosed LBD alone without AD diagnosis, we observe overall poor positive predictive values of only 34% (95% CI: 19%, 51%) to 47% (95% CI: 33%, 62%) at dementia stages CDR 3.0 and 2.0, respectively. Of these participants, the majority (32% at CDR 2.0 to 54% at CDR 3.0) harbored AD pathology, which was also indicated by Selvackadunco et al. (2019). This may negatively impact clinical trials that enroll LBD participants (Lee et al., 2019; Goldman et al., 2020).

A trend similarly observed for AD, LBD and AD+LBD is that when missed by the clinicians the most common alternative diagnosis after “No impairment” or “Undecided” is the Behavioral variant FTD syndrome or Primary progressive aphasia. While frontotemporal dementia is not the focus of our current study, our finding indicates potential over-diagnosis of this dementia variant at all levels of impairment.

Several recent studies which compare the clinical and pathological results are consistent with our results and may account for some our findings. They also indicate some directions to improve the clinical diagnostic accuracy for AD and LBD. One study (Surendranathan et al., 2020) demonstrated that patients with pathological LBD had a higher rate of receiving other alternative clinical diagnosis compared to non-LBD patients (46% vs. 13%), and it took a longer time for the clinical diagnosis to be made. This finding confirms our result that patients with LBD autopsy have a lower diagnosis accuracy compared with other groups even at later dementia stages. The results from Kurasz et al. (2022) demonstrate that Black/African American participants were less likely to receive LBD as the clinical primary diagnosis despite having a higher frequency of diffuse LBD pathology than their White and Hispanic counterparts. Another study (Chatterjee et al., 2021) noted that autopsy-confirmed AD+LBD had a premortem syndrome with higher prevalence of memory issues and less classical LBD symptoms compared to pure LBD pathology. Yet, AD+LBD presented with more parkinsonism, visual hallucinations, RBD and cognitive fluctuations than pure AD. Many of these studies (Surendranathan et al., 2020; Chatterjee et al., 2021; Kurasz et al., 2022) confirm that autonomic dysfunction (urinary incontinence, orthostatic hypotension) and repeated falls could be a significant diagnostic clue for LBD pathology (pure or mixed).

Our findings have implications on treatment considerations. That clinical Lewy body disease was more indicative of dual AD+LBD pathology may support the utility of cholinesterase inhibitors and memantine in this population (Wang et al., 2015), and also suggests that such patients may be candidates for amyloid-lowering therapies, which could be addressed in future clinical trials. Conversely, as pathologic AD+LBD was often clinically diagnosed as AD, this suggests that if AD patients display early psychosis, parkinsonism, REM sleep behavior disorder or dysautonomia, perhaps caution should be taken when using antipsychotics. Furthermore, if AD patients develop parkinsonism, other associated symptoms of clinical LBD above may be anticipated, thus warranting screening and specific treatment.

Finally, we highlight the results of the analysis on disparities of clinical diagnosis accuracy according to sex, race, age, and education years. Our results confirm previous studies (Husaini et al., 2003; Gianattasio et al., 2019; Lin et al., 2020; Alzheimer’s Association, 2021) by showing that for underrepresented groups (Females, Black, Older) the current clinical diagnostic performance is not sufficiently accurate at various stages of impairment. Improving the clinical evaluation instruments (Manly and Espino, 2004; Wood et al., 2006; Chin et al., 2011; Pedraza et al., 2012) and developing more accurate imaging and biofluid biomarkers particularly for underrepresented populations will be important steps towards better screening, diagnosis and treatment design for dementia. Seed amplification assays for alpha synuclein in cerebrospinal fluid (CSF), skin, and other biofluids and mucosa offer promising avenues to overcome these disparities by relying on biological evidence over clinical assessment, especially through routes that are less invasive (Bellomo et al., 2022). Similarly, more widespread use of PET and CSF measures of amyloid and tau, and, even more importantly, plasma measures of amyloid, tau, and other markers (e.g., NfL, GFAP) (Angioni et al., 2022) will offer more accurate assessment of AD co-pathology. At the same, more studies need to be done to assess how clinical syndromes may differ across demographic variables and how these variables impact LBD and AD biomarkers assessments, specifically with an examination of social determinants of health (Majoka and Schimming, 2021; Gleason et al., 2022).

Our study has some limitations: First, the cohort in our study is relatively small, primarily white and does not reflect the real-world population distribution. The small number of Black/African American participants might result in some selective bias (more willing to donate the autopsy for the research) to the statistical analysis. Second, when analyzing the diagnosis disparity for different groups, we only report the average metric over four subtypes (AD, LBD, AD+LBD, Neither). Further breakdown of each subtype was not possible due to the small sample size in our study. However, we acknowledge that the metrics reported likely will be different for different dementia subtypes. Third, other dementia subtypes (particularly vascular dementia and Frontotemporal dementia) are also important and necessary to study, however we only focused on AD, LBD, and AD+LBD in this study. We will expand similar analysis to other subtypes in future studies. Lastly, the dataset we used was focusing on AD research. In the clinic, diagnostic accuracy may be worse given less clinical information and less expertise of practitioners. Furthermore, the heterogeneity of cases will likely be higher in a clinic-based population. A research cohort may be biased to healthier participants less likely to have comorbidities that would further mask diagnostic accuracy.

## Data availability statement

Publicly available datasets were analyzed in this study. This data can be found here: https://naccdata.org/requesting-data/data-request-process.

## Ethics statement

Ethical review and approval was not required for the study on human participants in accordance with the local legislation and institutional requirements. The patients/participants provided their written informed consent to participate in this study.

## Author contributions

NR supervised the study and requested the data. HW and NR designed the study, analyzed the data, interpreted the results, drafted the manuscript, and verified the data. AM provided medical insights and edited the manuscript. All authors were responsible for the final submission.

## Footnotes

1 NPADNC in NACC Neuropathology data: https://files.alz.washington.edu/documentation/rdd-np.pdf.

2 NACCLEWY in NACC Neuropathology data: https://files.alz.washington.edu/documentation/rdd-np.pdf.

3 NACCALZD = 1 in NACC data: https://files.alz.washington.edu/documentation/uds3-rdd.pdf.

4 NACCLBDE = 0 or 8 in NACC data: https://files.alz.washington.edu/documentation/uds3-rdd.pdf.

5 NACCLBDE = 1 in NACC data: https://files.alz.washington.edu/documentation/uds3-rdd.pdf.

6 NACCALZD = 0 or 8 in NACC data: https://files.alz.washington.edu/documentation/uds3-rdd.pdf.

7 NACCALZD = 1 and NACCLBDE = 1 in NACC data: https://files.alz.washington.edu/documentation/uds3-rdd.pdf.

8 NACCETPR in NACC data: https://files.alz.washington.edu/documentation/uds3-rdd.pdf.

9 DECAGE in NACC data: https://files.alz.washington.edu/documentation/uds3-rdd.pdf. The cognitive decline durations are obtained by computing the difference between the age at the visit and the cognitive decline age. Only participants with the cognitive decline based on clinician’s assessment will be reported in the final results.
