# Supplementary material for: On gaps of clinical diagnosis of dementia subtypes: A study of Alzheimer’s disease and Lewy body disease
Source: Front Aging Neurosci. 2023 Mar 21;15:1149036. doi: 10.3389/fnagi.2023.1149036 (PMC10070837; doi:10.3389/fnagi.2023.1149036)
Supplement: Supplementary file 1 [file Table_1.DOCX]

Supplementary Material

On Gaps of Clinical Diagnosis of Dementia Subtypes: A Study of Alzheimer’s Disease and Lewy Body Disease

Hui Wei, Arjun V. Masurkar, Narges Razavian*

*** Correspondence:** Narges Razavian: narges.razavian@nyulangone.org

Table S1. Basic information about the NACC dataset used in the study

| **Total number of participants** | **The number of qualified participants** | **Time Span** | **NACC data set where the variables were collected** |
| --- | --- | --- | --- |
| 40,858 | 1,920 | September 2005 to August 2019 | The NACC Uniform Data Set (UDS)  The Neuropathology (NP) Data Set |

Table S2. Primary etiologic diagnosis names in the NACC dataset for Figure 4

| **Name in Figure 4** | **Name in the NACC dataset** |
| --- | --- |
| Undecided | Missing/unknown |
| Normal | Not applicable, not cognitively impaired |
| bvFTD or PPA | FTLD, other |
| Depression | Depression |
| Vascular | Vascular brain injury or vascular dementia including stroke |
| CBD | Corticobasal degeneration (CBD) |
| PSP | Progressive supranuclear palsy (PSP) |
| TBI | Traumatic brain injury (TBI) |
| Psychiatric | Other psychiatric disease |
| Prion | Prion disease (CJD, other) |
| Others 1 | Cognitive impairment for other specified reasons (i.e., written-in values) |
| Others 2 | Cognitive impairment due to systemic disease or medical illness |
| Others 3 | Cognitive impairment due to medications |
| Others 4 | Other neurologic, genetic, or infectious condition |
